# 20 Years of Legislation - How Australia Has Responded to the Challenge of Regulating Genetically Modified Organisms in the Clinic

**DOI:** 10.3389/fmed.2022.883434

**Published:** 2022-05-10

**Authors:** Gabrielle M. O’Sullivan, Joshua G. Philips, Heidi J. Mitchell, Michael Dornbusch, John E. J. Rasko

**Affiliations:** ^1^Research Ethics and Governance Office, Royal Prince Alfred Hospital, Sydney Local Health District, Sydney, NSW, Australia; ^2^Office of the Gene Technology Regulator, Australian Government Department of Health, Canberra, ACT, Australia; ^3^Department of Cell and Molecular Therapies, RPA Hospital, SLHD, Sydney, NSW, Australia; ^4^Faculty of Medicine and Health, The University of Sydney, Sydney, NSW, Australia; ^5^Gene and Stem Cell Therapy Program, Centenary Institute, The University of Sydney, Sydney, NSW, Australia

**Keywords:** gene technology, gene therapy, clinical medicine, gene technology regulation, risk management, technological and regulatory advances, ethics and law

## Abstract

• In contrast to the prior voluntary system, since 2001, gene technology in Australia has been regulated under a legislated national Gene Technology Regulatory Scheme which is administered by the Gene Technology Regulator.
• The Scheme provides science-based assessment of the potential risks of gene technology to the health and safety of people and the environment.
• It complements the role of the Australian Therapeutic Goods Administration which regulates all therapeutic products in Australia to ensure they are safe and effective.
• Recent reforms to the Scheme contribute to, and anticipate, the continued safe development and delivery of gene-based human therapeutics in Australia as a successful model for other jurisdictions.

## Introduction

Extraordinary progress in gene and cell therapies, and in new technologies for altering gene function has occurred over the last two decades. The pace at which these continue to enter mainstream clinical research and medicine is accelerating, and this increases community expectations on clinicians, regulators, manufacturers, and governments. The COVID-19 pandemic has further underlined the integral role that gene technology plays in the development of medicines and vaccines. It is therefore crucial that governments provide robust, but responsive, regulatory mechanisms to ensure safe and timely access to new therapeutics.

Australia regulates gene-based therapeutics *via* two inter-dependent routes: the *Gene Technology Act 2000*, which assesses the risks of gene technology to the health and safety of people and the environment; and the *Therapeutic Goods Act 1989*, which assesses the safety and efficacy of therapeutics for those who receive them. The review processes under these Acts are separate, independent, and complementary. The Australian regulatory system is unique in having a centralized regulator – the Gene Technology Regulator – dedicated to gene technology.

**FIGURE 1:**
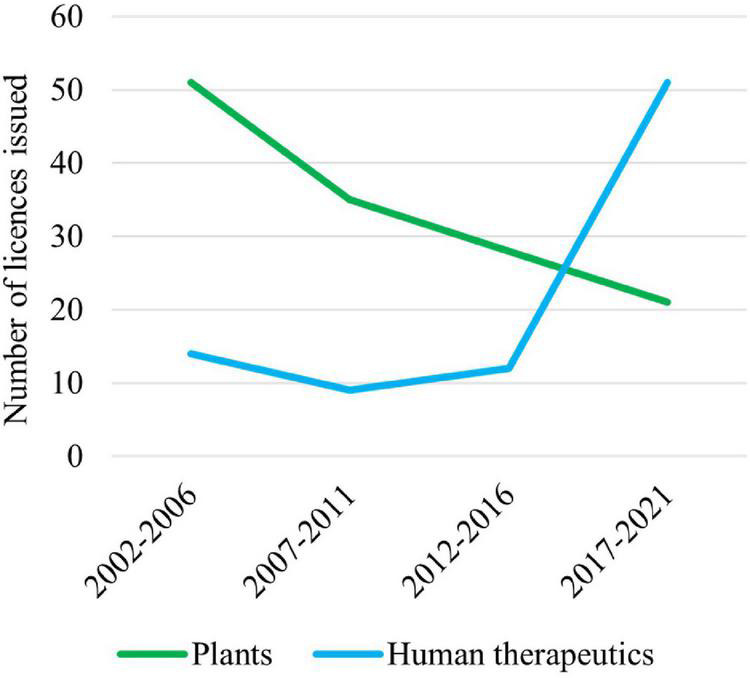
Licenses issued by the Gene Technology Regulator (the Regulator) for plants and use of human therapeutics involving GMOs over the last 20 years. Note that these data do not include licenses issued by the Regulator for research on vaccines and therapeutics that are confined to laboratories.

Australian Commonwealth Gene Technology legislation came into effect 20 years ago upon the commencement of the *Gene Technology Act 2000* and the Gene Technology Regulations (1) on June 21, 2001. This legislation underpins the *National Gene Technology Regulatory Scheme* (the Scheme). Initially, the majority of applications under the Scheme were for agricultural releases of genetically modified organisms (GMOs) or for research confined to laboratories, but the majority now involve human therapeutics (Figure 1).

**FIGURE 2:**
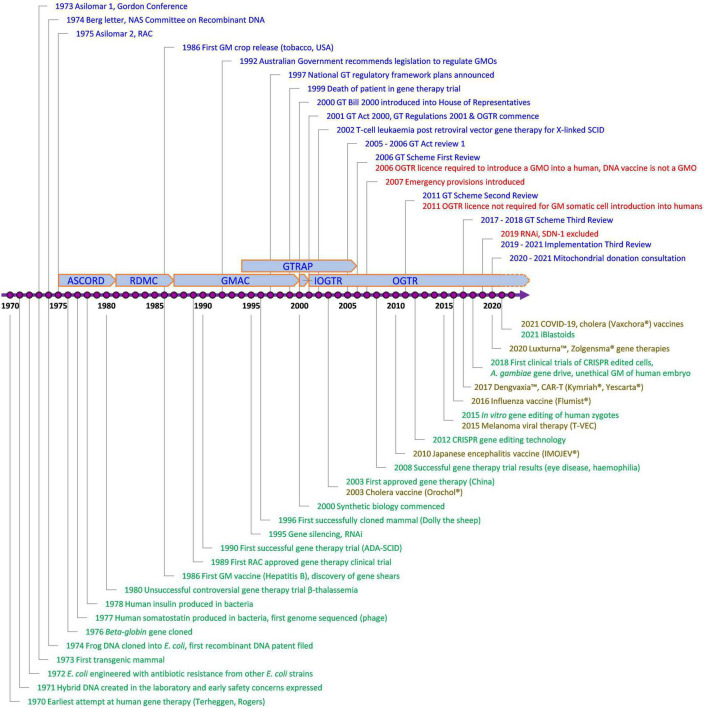
Key events involving gene technology and its regulation in Australia of relevance to human therapeutics. Blue, Contains key regulatory events; Green, Examples of key scientific or technological events; Red, Key changes to regulatory requirements as a result of amendments to the Australian Gene Technology legislation; Brown, Commercially approved gene based therapeutic products in Australia; ADA-SCID, Severe Combined Immuno-Deficiency caused by defective Adenosine deaminase gene; ASCORD, Australian Academy of Science Committee On Recombinant DNA; COVID-19, Coronavirus Disease 2019 caused by Severe Acute Respiratory Syndrome Coronavirus 2 (SARS-CoV-2); CRISPR, Clustered Regularly Interspaced Short Palindromic Repeats; DNA, Deoxyribonucleic acid; GM, Genetically Modified; GMAC, Genetic Manipulation Advisory Committee; GMO, Genetically Modified Organism; GT, Gene Technology; GTRAP, Gene and Related Therapies Advisory Panel; IOGTR, Interim Office of the Gene Technology Regulator; OGTR, Office of the Gene Technology Regulator; RAC, Recombinant DNA Advisory Committee, United States; RDMC, Recombinant DNA Monitoring Committee; RNAi, interfering RNA as technology; SCID, Severe Combined Immuno-Deficiency; SDN-1, Site-Directed Nuclease which does not involve the use of a guide nucleic acid. The earliest attempt at human gene therapy was an unsuccessful trial of wild-type *Shope papilloma virus* administered to three hyperargininemic subjects [Terheggen et al. (65)].

Since the Scheme began, technology has changed enormously, with major advances in gene and cell therapy, gene editing, synthetic biology, nanomaterials, personalized medicine, manufacturing, and delivery. Likewise, both globally and in Australia, there have been regulatory innovations to accommodate evolving technologies (Figure 2) and reforms to the Scheme have resulted from two public reviews (Table 1). The Third Review (2) is currently being implemented, and is the most wide-ranging as it aims to future-proof the Scheme. With the implementation of the Third Review and the 20th year anniversary of the Scheme, we reflect on the Scheme’s contribution to the safe development and application of gene technology in human therapeutics in Australia. We also examine how approval processes under the Scheme interact with other approval processes in Australia for therapeutic products. We discuss a number of challenges posed by gene technology and assess how implementation of the recommendations from the Third Review is expected to address them and optimize approval processes. We anticipate the information provided will be useful to sponsors contemplating regulatory strategies for gene-based therapeutics in Australia and to national and international policy makers in other areas that intersect with the impact of gene technology on humans.

**TABLE 1:** Australian regulatory reforms relevant to human gene-based therapeutics.

Review	Years	Object of reform	Status	Key reforms
First	2006	Regulations	Implemented	Explicit statement that an OGTR license is required to introduce a GMO into a human.
				A DNA vaccine is not a GMO. (Implemented 31 March 2007)
Second	2011	Regulations	Implemented	An OGTR license is not required to introduce a GM somatic cell therapy into a human. (Implemented 1 September 2011)
Third	2017-2020	Regulations	Implemented	Organisms treated using RNA interference or Site-Directed Nucleases (SDN) without guide nucleic acids (SDN-1) are not GMOs. (Implemented 8 October 2019)
		Scheme	In progress	Proposes a more risk proportionate regulatory framework that responds to technical advances.

## The Australian Gene Technology Regulatory Scheme

### Legislative Basis

The Scheme is a cooperative of all state, territory, and Commonwealth governments in Australia. It comprises the *Intergovernmental Gene Technology Agreement*, the *Gene Technology Act 2000* (the Act), the Gene Technology Regulations 2001 (the Regulations), and corresponding state and territory legislation. Prior to the Act, oversight of gene technology was under a voluntary guideline-based approach administered by the Genetic Manipulation Advisory Committee. The Act changed this to an enforceable legislated system under the Gene Technology Regulator (the Regulator) as an independent statutory office holder. The Regulator is responsible for administering the legislation in accordance with the object of the Act, which is to “*protect the health and safety of people, and to protect the environment, by identifying risks posed by or as a result of gene technology, and by managing those risks through regulating certain dealings with GMOs.*” The Regulator is supported by the Office of the Gene Technology Regulator (OGTR), which is located within the Commonwealth Department of Health. Two expert committees are available to the Regulator for advice if required: the *Gene Technology Technical Advisory Committee* (GTTAC) and the *Gene Technology Ethics and Community Consultative Committee* (GTECCC). Other regulators are required by law to consult the Gene Technology Regulator if a product under their remit also falls under the Gene Technology Act. Governance arrangements (3) facilitate the co-ordination and exchange of information between different regulators and stakeholders, while minimizing duplication. There is also capacity for the Australian Government minister responsible for gene technology to expedite the approval of dealings with a GMO in an emergency through an Emergency Dealing Determination (EDD). An EDD has effect for up to 6 months unless extended by the minister (4). As of 4 April 2022, there have been only two EDDs, both in 2007–2008 for GM vaccines for equine influenza.

### Entry to the Scheme Is Process Triggered

An organism is automatically regulated under the Scheme if it meets the definition of a GMO given in Section 10 (1) of the Act. A GMO is an organism that *“has been modified by gene technology”* or “*has inherited particular traits from an organism (the initial organism), being traits that occurred in the initial organism because of gene technology*,” unless it has been declared by the Regulations not to be a GMO. Thus, it is the application of the *process* of gene technology that triggers entry to the Scheme regardless of the outcome of the process. This contrasts with “*product*” triggered regulation in some other jurisdictions, such as Canada, where new organisms are regulated as “novel organisms” if their characteristics (traits) are considered to be new irrespective of the process by which they came about.

Importantly, although (other than for a gene modified cell therapy) a license is required from the Regulator to intentionally introduce a GMO into a human being, a human being who has received somatic cell gene therapy does not fall under the Scheme due to a specific exclusion in the Regulations. This exclusion only applies to human recipients of gene technologies.

### Risk Assessment

Similar to other gene technology regulatory schemes around the world, risk assessment under the Australian Scheme is science based, whereby the risk of a GMO is assessed against the risk of its unmodified parent organism. Formal assessments consider the potential for toxicity, allergenicity, replication competence, recombination, integration into host genomes, inadvertent transmission, and the impact of uncertainty regarding knowledge. Importantly, potential benefits - be they economic or health related - of the research are not considered in the assessment. The regulatory requirements are established in pre-defined classifications and legislated decision timeframes, and the Regulator’s assessments of DIR applications (see the section “Risk Management”) are publicly available. Together these ensure transparency, efficiency and predictability in the Scheme which the three public reviews have supported.

### Risk Management

Once an organism falls under the Scheme, the risk management requirements for working with it are tiered according to risk. For low risk contained laboratory-based research, the main authorization types are Exempt (or non-notifiable) and Notifiable Low Risk Dealings (NLRD). Higher risk dealings (for example, all *in vivo* viral vector human gene therapies) require a license from the Regulator. When assessing license applications, the Regulator performs a case-by-case risk assessment which, depending on the nature of the GMO and type of license, may require broad consultation with experts, other regulators, Australian state and territory governments and the public. This is to inform the Regulator’s decision on whether a license should be issued and what risk management conditions should be imposed in the license. Licenses fall into two categories: the first is a Dealing Not involving Intentional Release (DNIR) where the GMO is unlikely to be shed or dispersed into the environment; the second is a Dealing involving Intentional Release (DIR) of the GMO, where it is considered possible or probable that the GMO may be shed or dispersed into the environment. Commercial supply of therapeutic GMOs can fall under either DNIR or DIR categories. The Act also provides for the situation where a person comes into possession of a GMO without realizing or intending to. If this happens, all further dealings with the GMO, including destruction, require an authorization. In such cases, the Regulator may issue an inadvertent dealings or temporary license to facilitate the safe and legal disposal of a GMO (5). As of April 4, 2022, two such licenses have been issued, both for plants (GM petunia in 2017 and GM alfalfa in July 2021).

### Institutional Biosafety Committees, Human Research Ethics Committees, and the Therapeutic Goods Administration

The Scheme requires NLRDs and license applications to the OGTR to be reviewed by Institutional Biosafety Committees (IBCs). In line with the object of the Act and the OGTR, IBCs focus on managing risks to the environment, the health and safety of people working with GMOs, and others who may be unintentionally exposed to GMOs. As a result of the Scheme, most research institutions in Australia have in-house expertise in assessing and managing risks of GMOs.

The ethics, safety, and efficacy of treating human beings with therapeutics fall under the remit of Human Research Ethics Committees (HRECs) and the Therapeutic Goods Administration (TGA). The TGA authorizes clinical trials of “unapproved” therapeutics and approves therapeutic goods for inclusion on the Australian Register of Therapeutic Goods (ARTG). Inclusion on the ARTG means the therapeutic goods can be lawfully supplied in Australia.

Clinical trials must be reviewed by HRECs before being sent to the TGA as a Clinical Trial Notification (CTN) or as a Clinical Trial Authorisation (CTA) application. CTNs make up over 99% of submissions to the TGA and, as notifications, are not subject to review by the TGA after HREC review. CTAs are required for higher risk or novel treatments, such as Class 4 biologicals (which include GM cell therapies) and are subject to TGA review.

Although the OGTR and TGA are two separate competent authorities responsible for the GMO and clinical aspects, respectively, they communicate with each other and with other regulators (for example, the US Food and Drug Administration and the World Health Organization) where warranted. Similar to many other countries, applications to both authorities are not linked and may be submitted in parallel, although GMO licenses need to be issued before the clinical trial can commence.

Together, these processes are designed to provide protection of human health and safety and the environment at all stages in the development and clinical application of therapeutic GMOs.

## Gene Technology in Human Therapeutics

### Current Gene Technologies Used in Clinical Medicine

Gene technology is designed to modify existing genes (or gene expression) or deliver new or missing genes into human cells for gene therapy for therapeutic benefit. Gene technologies are also used in other organisms for vaccine production. The delivery vehicles are often derived from existing organisms (known as “parent organisms”) by attenuating or removing potentially harmful genes from their genomes and introducing therapeutic modifications into them. Such vehicles include replication defective viral vectors (such as gene therapy vectors derived from lentivirus or *Adeno-associated virus* (AAV), which have had most of their viral genome removed), attenuated replication competent viruses (such as *Herpes simplex virus-1* or *Vaccinia virus* vaccine strains modified to selectively replicate in, and lyse, tumors in the case of cancer therapies, or to generate a protective immune response in the case of vaccines) and bacteria (such as *E. coli* genetically engineered to restore antibiotic susceptibility to gut bacteria).

**FIGURE 3:**
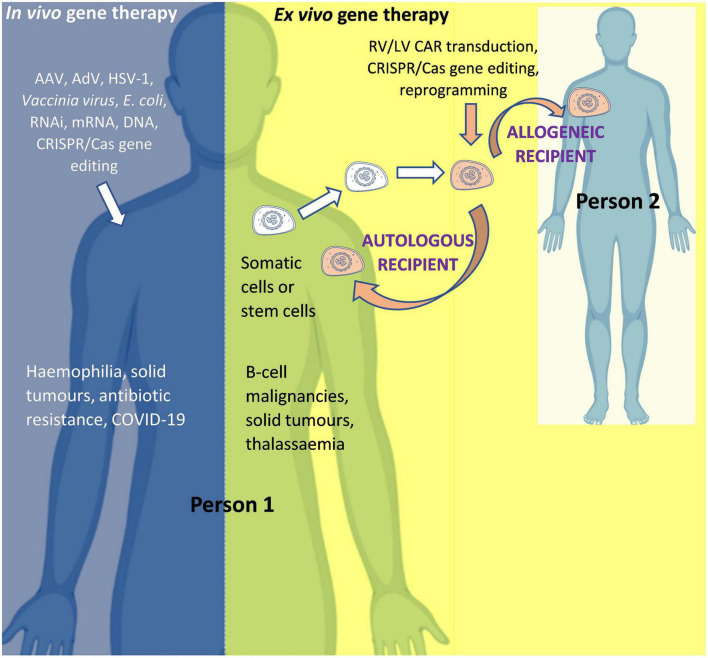
*In vivo* and *ex vivo* gene therapy. AAV, *Adeno-associated virus*; AdV, *Adenovirus*; Cas, CRISPR associated protein; CAR, Chimeric Antigen Receptor; COVID-19, Coronavirus Disease 2019 caused by Severe Acute Respiratory Syndrome Coronavirus 2 (SARS-CoV-2); CRISPR, Clustered Regularly Interspaced Short Palindromic Repeats; HSV-1, *Herpes simplex virus 1*; LV, Lentiviral vector; mRNA, messenger RNA; RNAi, RNA interference; RV, Retroviral vector. Person 1 is the patient if the product is for (“autologous”) use in that same person OR is a healthy donor if the product is for (“allogeneic”) use in another person (Person 2). For *in vivo* gene therapy the modifying gene transfer vector is directly introduced into the body. In *ex vivo* gene therapy, stem cells (such as hematopoietic stem cells for the treatment of thalassemia) or immune system cells (such as T-cells for the treatment of cancer) can be isolated from the body, modified, and then re-introduced into the body. An allogeneic therapeutic product is manufactured from the biological material of a person other than the patient (as the donor). It can be manufactured for a specific patient under the responsibility of a medical practitioner (as a “directed allogeneic use” product) or for many patients (as an “off-the-shelf” product).

The two main routes of administration used to modify genes or gene expression in human gene therapy are: *in vivo gene therapy*, whereby the modifying agent is administered directly into the human body (for example AAV-based gene therapy for hemophilia); and *ex vivo gene therapy*, whereby cells are collected from a donor and then modified in a specialized laboratory before being administered to a recipient (for example, chimeric antigen receptor (CAR) T-cell therapy for cancer). Although both are forms of gene therapy, the former (*in vivo*) is often referred to as “gene therapy” and the latter as “*ex vivo* gene therapy,” “gene modified (GM) cell therapy,” or simply “cell therapy.” For *ex vivo gene therapy*, if the donor and recipient are the same person, the therapy is known as “autologous”; and if the donor and recipient are different, the therapy is known as “allogeneic” (Figure 3).

While virus- and viral vector-based vehicles enter cells by binding cellular receptors (“transduce”), nucleic acids may be introduced in the absence of viruses or viral vectors. Non-viral vectors include deoxyribonucleic acid (DNA), ribonucleic acid (RNA), and messenger RNA (mRNA), which may be “naked” or linked to lipids or other chemical entities to facilitate entry.

Other relevant technologies include RNA interference (RNAi) and gene editing. RNAi involves the use of small RNA molecules to interfere with (“silence”) gene expression by several different non-exclusive molecular mechanisms. Gene editing using CRISPR/Cas is a recently established and advancing technology that promises to achieve precise edits to the genome. The CRISPR/Cas components can be delivered into cells in a variety of ways (e.g., by expression from viral vectors, non-viral plasmids and mRNA or the Cas protein introduced directly) and can be administered *in vivo* or *ex vivo* depending on the therapeutic goal (6, 7).

### Clinical Applications of Gene Technology

Globally, many vaccines are manufactured using gene technology. Human gene therapy is a more recent development spurred by the successful treatment, in September 1990, of a patient in the United States with a gene modified cell therapy for severe combined immunodeficiency (SCID) due to adenosine deaminase deficiency (8). Unfortunately, in 1999 a participant died in a gene therapy trial for ornithine transcarbamylase deficiency (9, 10). This led to a suspension of research at the host institution, a major investigation, and a tightening of regulatory oversight. Later, the development of leukemia in patients treated with a gene modified cell therapy for X-linked SCID from 2000 to 2002 (11–14) resulted in a halt to all similar gene therapy trials in the United States and Europe from 2002 to 2003. However, from 2007 onward safer vectors became available (15, 16), and successes were achieved in clinical trials for inherited retinal disease (17), hemophilia (18–21), β-thalassemia (22), sickle cell disease (23), B-cell lymphoma (24) and B-cell leukemia (25–28). Product marketing authorizations have now been achieved in various jurisdictions for a number of gene-based therapies such as, for example, Novartis’ Luxturna^®^ for vision loss, bluebird bio’s LentiGlobin^®^ BB305 for β-thalassemia (Zynteglo), Novartis’ Kymriah^®^ for B-cell acute lymphoblastic leukemia, Gilead Kite’s Yescarta^®^ for specific types of lymphoma (29, 30), and Novartis’ Zolgensma^®^ for spinal muscular atrophy (31).

**FIGURE 4:**
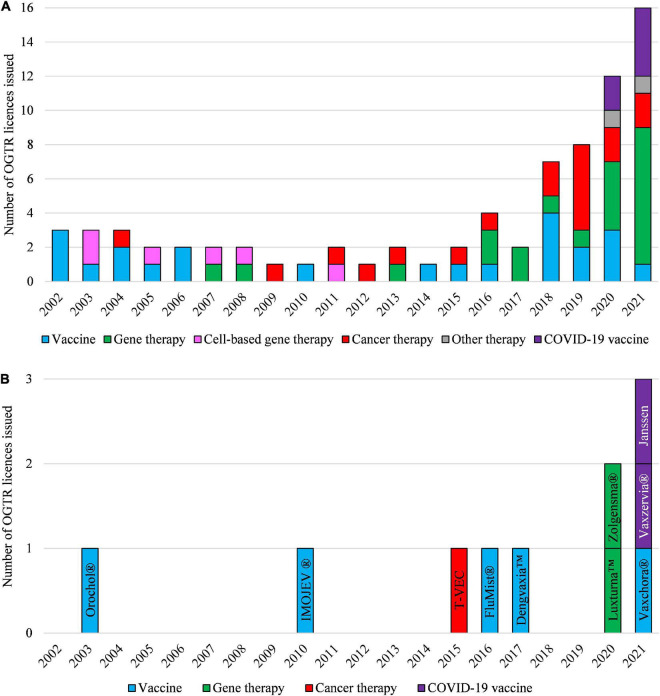
Types of OGTR licenses issued for human clinical trials with GMOs **(A)** and for commercial therapeutic use of GMOs **(B)** over the last 20 years. **(A,B)** The year of an OGTR license issue may not have been in the same year that an application to conduct use of the GMO was received. Cell-based therapy (pink shading, **A**) ceased to be regulated by the OGTR in 2011. **(B)** Key: Orochol (Cholera vaccine, live oral); IMOJEV (*Japanese encephalitis* vaccine, live, attenuated); T-VEC (IMLYGIC, Talimogene laherparepvec); FluMist (Influenza vaccine); Dengvaxia (Dengue tetravalent vaccine, live); Luxturna (Voretigene neparvovec); Zolgensma (onasemnogene abeparvovec); Vaxchora (Cholera vaccine, live oral); Vaxzervia (COVID-19 vaccine, AstraZeneca); Janssen (COVID-19 vaccine, Janssen). For further information and details of these data see Supplementary Tables 1, 2.

**FIGURE 5:**
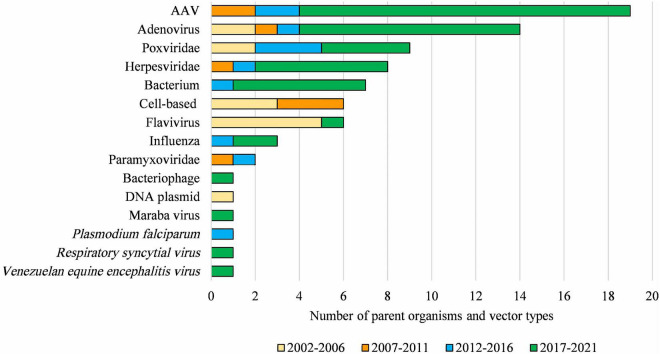
Diversity of parent organisms and vector types used in licensed clinical trials from 2002 to 2021. AAV, *Adeno-associated virus* of various serotypes; *Adenovirus*, includes various human, chimpanzee, and ovine serotypes; Poxviridae, includes various strains of *Vaccinia virus* and *Fowlpox virus*; Herpesviridae, includes *Herpes simplex virus 1* and *Human cytomegalovirus*; Bacterium, includes *Bifidobacterium longum, Bordetella pertussis*, *Listeria monocytogenes*, *Mycobacterium bovis and Vibrio cholera;* Flavivirus, includes Yellow Fever and Dengue viruses; Paramyxoviridae, includes *Bovine parainfluenza virus* and *Sendai virus*. DNA-based vaccines and cell-based therapies ceased to be regulated by the OGTR in 2007 and 2011, respectively. This Figure does not include parent organisms in commercial licenses. For further information and details for these data see Supplementary Table 1.

Clinical applications, and the regulatory status, of gene technology in Australia from 2002 to 2021 are reflected in the number and types of OGTR licenses granted for clinical trials (Figure 4A) and commercial therapeutic releases (Figure 4B), the diversity of parent organisms used (Figure 5), the examples of gene-based therapies (Table 2) and in the case-studies presented below.

**TABLE 2:** Exemplars of gene-based therapies, their regulatory status, and regulatory requirements in Australia.

**Vaccines**	**Biocelect Vaxchora (PXVX0200) (previously Orochol)**	**AstraZeneca FluMist**
Indications	Cholera vaccine	Influenza vaccine
Type	GM live attenuated *Vibrio cholerae* virus vaccine strain	GM live attenuated human *Influenza A* and *B* virus vaccine strains
Administration	Per oral	Nasal spray
Clinical status	Authorized: United States, EU; Pending authorization: Australia	Authorized: United States, Canada, EU, Australia
OGTR license	Yes (DIR-174 for commercial supply)	Yes (DIR-137 for commercial supply)

**Gene therapy**	**Spark Therapeutics SPK-8011, Pfizer SPK-9001 (PF-06838435, Fidanacogene elaparvovec)**	**Novartis Luxturna (Voretigene neparvovec)**
Indications	Hemophilia A (SPK-8011), Hemophilia B (SPK-9001)	Inherited blindness due to *RPE65* gene mutations
Type	GM replication deficient AAV vector expressing clotting factors VIII for Hemophilia A or IX for Hemophilia B.	GM replication deficient AAV vector expressing human retinal pigment epithelium 65 kDa (RPE65) protein
Administration	Single IV infusion	Subretinal injection
Clinical status	Clinical studies	Authorized: EU, United States, Switzerland, Australia, Canada
OGTR license	Yes (DNIR-569 and DNIR-577 for clinical studies)	Yes (DNIR-615 for commercial supply)

**Cell therapy**	**LentiGlobin BB305 Drug Product**	**CRISPR Therapeutics CTX110, CTX120, CTX130**
Indications	Transfusion-Dependent β-Thalassemia (TDT), Sickle Cell Disease (SCD)	CD19^+^ B-cell malignancies; BCMA^+^ multiple myeloma; CD70^+^ solid tumors
Type	GM autologous CD34^+^ HSC modified *ex vivo* with a replication defective, self-inactivating, lentiviral vector encoding functional β-globin	GM allogeneic healthy donor T-cells gene edited *ex vivo* using CRISPR/Cas9 to insert chimeric antigen receptor (CAR) genes targeting cancer-specific antigens: CD19 (CTX110), BCMA (CTX120) and CD70 (CTX130)
Administration	Single dose IV infusion	Flexible dosing IV infusion
Clinical status	Conditionally authorized for TDT in EU (Betibeglogene autotemcel, Zynteglo). Clinical studies for SCD in United States (NCT02140554). Then voluntary temporary suspension February – July 2021 while pending outcome of a safety review by EMA in 2021, which “concluded that there is no evidence Zynteglo causes a blood cancer known as acute myeloid leukemia” (64).	Clinical studies
OGTR license	Not required (Somatic Cell therapy)	Not required (Somatic Cell therapy)

**Cancer**	**Amgen IMLYGIC (Talimogene laherparepvec, T-VEC)**	**CG Oncology CG0070**
Indications	Melanoma (unresectable)	Bladder cancer due to defects in retinoblastoma (Rb) signaling
Type	GM live attenuated replication competent virus (*Herpes simplex virus-1*, JS1) modified to express *hGM-CSF* to enhance systemic anti-tumor immune responses and oncolysis	GM live attenuated replication competent virus (human *Adenovirus*) modified to preferentially replicate in cancer cells with defects in Rb signaling and express *hGM-CSF* to enhance systemic anti-tumor immune responses and oncolysis.
Administration	Multiple treatments *via* intra-tumoral injections	Weekly treatments *via* intravesical (IVE) route
Clinical status	Authorized: United States, EU, Australia	Clinical studies
OGTR license	Yes (DIR-132 for commercial supply)	Yes (DIR-177 for clinical studies)

**Other**	**Westmead Institute for Medical Research GM *E coli* to restore antibiotic sensitivity to gut bacteria**	**Prevail Therapeutics PR006**
Indications	Reduced effectiveness of certain medical treatments affected by antibiotic resistance in gut bacteria	Frontotemporal dementia with pathogenic *progranulin gene* (*GRN*) mutations
Type	*E. coli* (Nissle) containing antibiotic resistance plasmids with genes for resistance to multiple antibiotic classes deleted to restore antibiotic sensitivity to gut bacteria	Replication defective AAV vector encoding human progranulin protein (PGRN)
Administration	Ingestion	Single dose *via* intra-cisternal administration
Clinical status	First-in-human clinical study (pending HREC/TGA)	First-in-human clinical study
OGTR license	Yes (DIR-183 for clinical study)	Yes (DNIR-623 for clinical study)

**COVID-19**	**VAXZEVRIA (Previously AstraZeneca ChAdOx1-S, AZD1222, ChAdOx1 nCoV-19)**	**Pfizer COMIRNATY [BNT162b2 (mRNA)]**
Indication	COVID-19 vaccine	COVID-19 vaccine
Type	Replication defective Chimpanzee Adenovirus type Oxford University 1 (ChAdOx1) vaccine vector encoding SARS-CoV-2 spike protein	Non-replicating single stranded nucleoside-modified messenger RNA (mRNA) encoding SARS-CoV-2 spike protein
Administration	2 doses *via* intramuscular injection	2 doses *via* intramuscular injection
Clinical status	Authorized: Many countries, ongoing safety assessments	Authorized: Many countries, ongoing safety assessments
OGTR license	Yes (DIR-180 for commercial supply, DNIR-630 and DNIR-632 for manufacture)	Not required (mRNA)

*AAV, Adeno-associated virus; BCMA, B-cell maturation antigen; Cas9, CRISPR associated protein 9; CAR, Chimeric Antigen Receptor; CAR-T, Chimeric Antigen Receptor T Cells; COVID-19, Coronavirus Disease 2019 caused by SARS-CoV-2; CRISPR, Clustered Regularly Interspaced Short Palindromic Repeats; DIR, OGTR License for a Dealing Involving Intentional Release; DNIR, OGTR License for a Dealing Not Involving Intentional Release; hGM-CSF, human Granulocyte-Macrophage Colony-Stimulating Factor; HSC, Hematopoietic Stem Cells; IV, Intravenous Infusion; SARS-CoV-2, Severe Acute Respiratory Syndrome Coronavirus 2. “Clinical status” refers to whether the therapy is in clinical studies or is authorized (i.e., has received marketing approval). “Authorized” (for each jurisdiction) means marketing approval has been granted by the following (for example only): Provisional Approval and entry onto the Australian Register of Therapeutic Goods (ARTG) (Australia), Provisional Consent (New Zealand), Conditional Marketing Authorization (EU), FDA License (Approval) (United States), National Institute for Health and Care Excellence (NICE) approval (United Kingdom). All authorizations are subject to ongoing safety assessment and reporting.*

The first license for a clinical trial (DNIR-071) was issued by the Regulator in 2002 to the Australian Defense Force Malaria and Infectious Disease Institute for a viral vector-based Japanese encephalitis vaccine. The first licenses for *ex vivo* gene modified cell therapy trials were issued in 2003 to Johnson & Johnson Research Pty Ltd., (DNIR-170) for autologous CD34^+^ hematopoietic progenitor cells (HPC) transduced with a retroviral vector containing an anti-HIV-1 ribozyme, and The Children’s Hospital at Westmead (DNIR-179) for autologous CD34^+^ HPC transduced with a retroviral vector containing genes to provide resistance to alkylating drugs used in cancer therapy. The first commercial release was licensed in 2003 to CSL Ltd., (DIR-033) for the Cholera vaccine Orochol^®^, and the first *in vivo* gene therapy clinical trial license was issued to The University of Western Australia in 2007 for an AAV trial for age-related macular degeneration (DNIR-415).

Regulatory reforms have since removed some types of human therapeutics from regulatory oversight as gene technology or removed the requirement for a license (Table 1). These include DNA vaccines (removed 31 March 2007), GM somatic cell therapy (removed 1 September 2011, whereby (as mentioned previously) clinical trials of GM cell therapies such as those previously covered under the 2003 licenses DNIR-170 and DNIR-179, and CAR T-cell therapies, no longer required OGTR licenses), and RNAi and Site Directed Nucleases without guide RNAs (removed October 8, 2019).

Much of the research on vaccines and therapeutics takes place in laboratories and is therefore not within the scope of this clinically focused review. Furthermore, regulatory reforms were implemented on 1 September 2011, whereby clinical trials of GM somatic cell therapies, such as CAR T-cell therapies, have not required an OGTR license. In addition, *in vitro* handling of GM cells in the laboratory was classed as an exempt GMO dealing so that such studies now only require review by IBCs, HREC’s, and the TGA (not the OGTR).

Therefore, the data presented herein after 2011 do not reflect the therapeutic implementation of many GM-based medicines. However, it is widely accepted that regulatory reforms have been instrumental in attracting GM somatic cell therapy clinical trials to Australia.

## Case Studies

### Hemophilia

Without treatment, hemophilia is a debilitating inherited bleeding disorder caused by mutation or deletion in the gene for clotting factor 8 in the case of hemophilia A, or 9 in hemophilia B. This results in bleeding into joints and greatly affects patients’ quality of life. It can be life threatening and simple activities such as playing sport, going on holidays, or undertaking manual work can be very challenging. A number of successful hemophilia A and B gene therapies have resulted from research initiated by the Children’s Hospital of Philadelphia (CHOP) using AAV (32, 33), and by University College London and St. Jude Children’s Hospital (19). Royal Prince Alfred Hospital in Sydney participated in the earliest clinical studies with CHOP (18, 19), and obtained the first OGTR license for AAV gene therapy for hemophilia (hemophilia B) in Australia in 2008. Since then, other licenses for AAV hemophilia gene therapy have been issued to Pfizer, BioMarin and Medpace, and trials have been conducted at other hospitals around Australia. Subjects have greatly benefited as the studies have “*enabled the termination of baseline prophylaxis and the near elimination of bleeding and factor use”* (20, 34). For example, therapeutic levels of clotting factor and a 91% reduction in bleeding rates have been demonstrated over 3 years for hemophilia A (21).

Between 2008 and 2016, when the early OGTR licensed hemophilia AAV gene therapy studies were underway in Australia, and local viral vector gene therapy clinical studies remained relatively infrequent, awareness and risk management was not streamlined across the country. The legally enforceable conditions in early OGTR licenses ensured staff were trained in risk management and helped to facilitate requirements nationally. The licenses also help hospitals manage risk because the license holders (usually commercial sponsors) are held responsible for compliance. OGTR mandated Compliance Management Plans for each license further ensure that sponsors apply consistent risk management standards across multiple trial sites and consult the local IBCs before studies begin. A key problem has been that, because Australia does not have a substantial pharmaceutical manufacturing sector, local sponsors (frequently marketing and clinical trial branches of international sponsors) do not often have the required technical knowledge to complete OGTR license applications. They have frequently required back and forth consultations with technical and regulatory experts in the parent company. IBCs have to educate sponsors on Australian requirements and technical aspects which has consequently delayed approvals. This is further exacerbated if the parent company is large and has unwieldy top-down or siloed communication and authorization processes that are not well suited to biological therapies.

### Vaccines and Public Health

Initially, live vaccines were attenuated using techniques such as serial passage. Now this can be achieved by genetic modification (for example, by removing viral replication and immune evasion genes) and GMOs are increasingly being used as attenuated live-vaccines or to produce subunit vaccines.

In Australia, the Regulator has issued licenses for clinical trials of vaccines for *Respiratory syncytial virus*, influenza, cholera, malaria, whooping cough, COVID-19, *Cytomegalovirus*, Hepatitis B, and Zika/Chikungunya viruses. Commercial supply licenses have been granted for vaccines against Japanese encephalitis (IMOJEV^®^, Sanofi, 2010), influenza (FluMist^®^, AstraZeneca, 2016), Dengue fever (Dengvaxia^®^, Sanofi, 2017), cholera (Orochol^®^, CSL, 2003; Vaxchora^®^, Biocelect, 2021) and COVID-19 (Vaxzervria^®^, AstraZeneca, 2021; COVID-19 vaccine, Janssen, 2021).

Researchers and companies across the world have been urgently developing candidate vaccines for coronavirus disease 2019 (COVID-19) caused by the SARS-CoV-2 virus. As of 1 April 2022, the World Health Organization reported 153 vaccines in clinical development and 196 in preclinical development globally (35). Regulators have responded in parallel by providing timely assessments without compromising safety.

Without need for regulatory adjustment, the regulatory status of dealings with vaccines in Australia is clear under the current Scheme. Dealings involving GM virus or viral vector-based vaccines (such as AstraZeneca’s and Janssen-Cilag’s adenovirus-based COVID-19 vaccines) or GM bacteria-based vaccines require OGTR licenses. In contrast, mRNA or protein sub-unit vaccines (such as Pfizer’s COMIRNATY™ and Moderna’s mRNA-1273 mRNA COVID-19 vaccines, and Novavax’s NVX-CoV2373 protein sub-unit COVID-19 vaccine) do not require a license because mRNA and proteins derived from the use of gene technology are not defined as GMOs under the Scheme. This is because GM viruses, viral vectors and bacteria have the potential to replicate, spread, or cause harm to the environment or people handling them unless they are manufactured, contained, and used appropriately. In contrast, mRNA and proteins do not have this potential.

Where COVID-19 vaccines have required an OGTR license, the OGTR has expedited its assessments to precede, or align with, TGA product approvals. Thus, COVID-19 vaccines have not required exemptions from any gene technology related regulatory requirements in Australia. In this way, the national consistency of the Scheme, with the OGTR as its central expert regulatory competent authority for GMO aspects, and its network of experienced IBCs ensures Australia assesses GM vaccines in a safe and timely way.

## Discussion – Key Challenges and How Recommendations of the Third Review are Expected to Address them

The key challenges facing the future development and use of gene-based therapeutics relate to technological advances, risk proportionate regulation, harmonization with other regulatory systems, access, the patient journey, and marketability. They are inter-related and impact society, the environment, ethics, and safety in the broadest sense. It follows that regulatory systems need to be able to respond to rapidly changing needs and interact well with each other.

### Technological Advances

**FIGURE 6:**
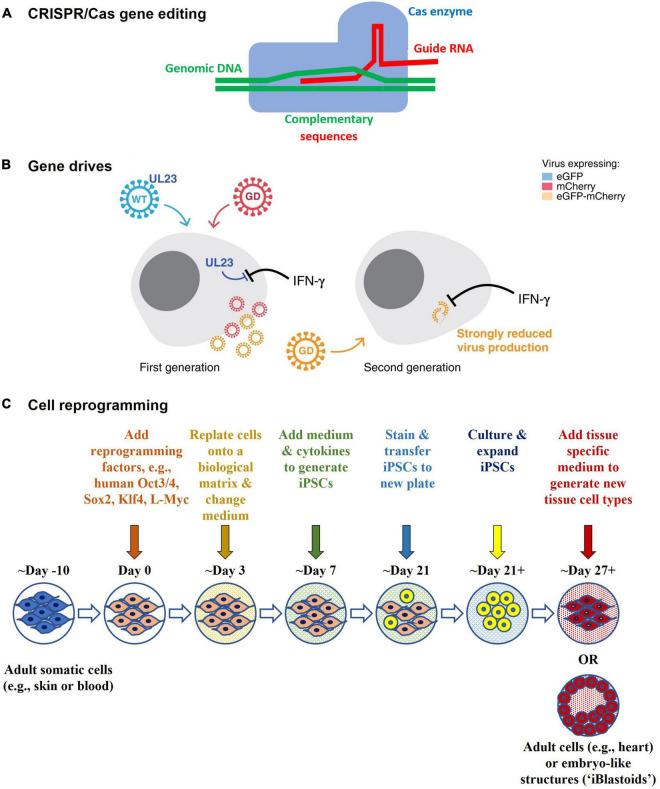
Technological advances **(A)** CRISPR/Cas gene editing – CRISPR/Cas comprises an enzyme (Cas), which is complexed with a synthetic guide RNA that directs the enzyme to a target site in the genome where it cleaves specific DNA sequences and allows sequences to be added, removed, or altered *in situ* (i.e., “edited”). Cas9 is the first gene editing enzyme developed by Emmanuelle Charpentier and Jennifer A. Doudna in 2012, for which they received the Nobel Prize in Chemistry in 2020. Other types of editors have been developed to provide greater safety, functionality, and finer control over gene editing. **(B)** Gene drives – Gene drives are genetic elements that are favored for inheritance. They increase the rate at which certain genes are inherited by the offspring of reproducing organisms, thus spreading the genes faster through a species than would normally occur. They can be used to preferentially propagate chosen genetic modifications in a target population, even if deleterious to the population. They can be generated in organisms that reproduce sexually (e.g., mosquitos for malaria control) or asexually (e.g., bacteria [*E. coli*] and viruses [Human cytomegalovirus (HCMV)]), and may have potential for infectious disease control. The schematic is from Walter and Verdin (61) and shows how a gene drive (“GD”) in HCMV might be used to target and replace wildtype HCMV (“WT”) in cell culture experiments. The WT expresses UL23 which blocks interferon-gamma (IFN-γ) antiviral responses, whereas in the GD UL23 is knocked out, thus making the GD susceptible to IFN-γ. In addition, they each express a different marker protein that enables them to be distinguished from each other *via* fluorescence microscopy. The WT expresses a green fluorescent marker protein (eGFP), whereas the GD expresses a red fluorescent marker protein (mCherry). Recombination between the WT and GD gives rise to recombinant GD + viruses that are strongly inhibited by IFN-γ when infecting other cells and that express both marker proteins (eGFP-mCherry). **(C)** Cell reprogramming - Mature (adult somatic) cells can be reprogrammed in the laboratory to an immature state (as induced pluripotent stem cells, iPSCs) by treating them with reprogramming factors. They can then be differentiated into other types of mature cells. For example, an adult skin cell can be reprogrammed to become a heart muscle cell. It may not be necessary to go through the stem cell state, as direct reprogramming from one type of mature cell (e.g., skin) to another (e.g., heart) is possible in the laboratory. Reprogramming factors may be introduced into cells using gene technology (such as *via* viral vector or plasmid transduction) or without gene technology (such as *via* chemical protein induction). The Polo laboratory at Monash University generated human embryo-like structures (“iBlastoids”) from adult skin cells using such processes (57–59).

Technology has advanced to a point where possibilities once considered likely, but not imminent, have become real; particularly following the establishment of programmable gene editing technology *via* targetable nucleases and CRISPR/Cas (36, 37) (Figure 6). Two striking examples of this are the *in vitro* gene editing in 2015 of human tripronuclear zygotes not intended for implantation to establish a pregnancy (38), and the announcement in November 2018 of widely condemned unethical research involving gene editing of human embryos that were subsequently implanted to establish a pregnancy (39). The latter has revived earlier considerations of, and provoked renewed international statements on, the ethics of Human Inheritable Genetic Modification (HIGM) (40–43); as well as considerations of the adequacy of current oversight mechanisms regarding the potential for HIGM. These concerns are also apparent in Australia (44–47). The Third Review recommended that “*subject to consideration, the COAG (Council of Australian Governments) Health Council might also consider whether additional regulatory oversight is needed for humans who may receive or inherit germline therapies (or other somatic therapies not within the remit of the Scheme). The COAG Health Council should also consider which regulatory (or other) body would be most appropriate to undertake such oversight”* (44). In another sphere of public policy development, consultations on the legalization of mitochondrial donation considered the potential for intentional HIGM (45, 46), and as a result the *Mitochondrial Donation Law Reform (Maeve’s Law) Bill* (48), which was passed on 30 March 2022 expressly prohibits intentional modification of nuclear or mitochondrial DNA. Once legalized, mitochondrial donation will be regulated by the National Health and Medical Research Council Embryo Research Licensing Committee.

Other indicators of future directions are provided in the clinic and in the laboratory. In the clinic, they include direct *in vivo* human gene editing for Transthyretin Amyloidosis (7) and the treatment of graft versus host disease using reprogrammed cells (49). In the laboratory, they include the creation of new organisms using synthetic biology (50–54) (such as *Horsepox virus* (55) in research aimed at developing safer vaccines) and the reprogramming of somatic cells (49, 56) from a mature state into a pluripotent stem cell state, and into human embryo-like structures (“iBlastoids”) (57–59) for the purpose of elucidating disease mechanisms and new therapeutics. Gene drives have also been developed as a potential means for infection control, such as in mosquitoes for the control of malaria (60), and in *Human Cytomegalovirus in vitro* in proof of principle experiments (61). Synthetic biology and gene drives are within the scope of the current Scheme and there is strong support for this to continue (44).

Changes in technology incorporate advances in personalised medicine, synthetic materials, nanomaterials as delivery and activation agents, biological materials and cells as devices, manufacturing using automated closed systems (whereby devices, not facilities, provide containment of GMOs), and flexible manufacturing and delivery processes (including at point-of-care) that respond to patients’ needs in real-time. Implementing these technical developments require ongoing communication between the OGTR and TGA.

Broader participation in science outside traditional research organizations is important and the OGTR regulates, actively engages, and provides advice in this area. Importantly, the Scheme has also enabled Australian community attitudes to gene technology to be gauged regularly. The most recent report in June 2021 found that there is “*stronger support for genetic modification generally at 39% of high support in 2021, up from 33% in 2019*”; and that “*genetic modification for medical purposes remains the most acceptable use, with strong support at 61% of people surveyed*” (62).

### Risk Proportionate Regulation, Harmonization With Other Regulatory Systems and Marketability

At present, the Scheme consists of prescriptive or rules-based regulations that can only be changed *via* legislation. Advantages of the Scheme, as it currently stands, are that it provides “*full regulatory coverage of gene technology across Australia,”* rigor, clarity, and certainty. One obvious disadvantage is that rules-based regulations can “*lack the agility needed to keep pace with the advances in technology”* (44). The Third Review recognized this and aims to future-proof the Scheme by providing a means for additional risk tiering, and principles-based legislation with supporting delegated legislation. Principles-based regulation sets out high-level principles that focus more on outcomes than on the means of achieving them. Some rules-based regulation can be retained for clarity where needed and delegated legislation can enable regulators to make changes in response to new information without having to change the underlying legislation.

Research, development, commercialization, and the regulation of gene technology are global activities, and regulatory harmonization is vital to ensure the flow of goods and services across borders. Although definitions and approval processes for therapeutic applications of gene technology may differ, risk management approaches are similar between jurisdictions. However, terminology matters, and much remains to be achieved in harmonizing terminology between countries so that therapeutic goods can flow freely. For example, while the Australian “exempt GMO” classification applied to a therapeutic (such as therapeutics derived from an induced pluripotent stem cell that originated using gene technology) is not problematic in Australia, it could be problematic for export to other jurisdictions if the same therapeutic is considered and marketed as a non-GMO. Implementation of the recommendations in the Third review are expected to provide greater flexibility in dealing with issues such as this with flow-on benefits toward improved global harmonization. The OGTR contributes to harmonization and best regulatory science practices through its interactions with other regulators in Australia *via* the Regulatory Science Network, and internationally by its participation in the OECD, WHO and other multilateral forums. The TGA also participates in many international regulatory harmonization activities.

### Access, Timeframes, International Awareness of Regulatory Requirements in Australia, and the Patient Journey

Increased harmonization and accelerated timeframes for all types of regulatory approvals have been identified as important factors impacting patient access to therapeutics in the recent House of Representatives “Inquiry into approval processes for new drugs and novel medical technologies in Australia” (63). While TGA and OGTR target timeframes align with each other, and with international regulators, for product approvals (approximately 120–255 business days for TGA product approvals and 90 – 255 business days for OGTR licenses), there is a divergence between the OGTR and TGA in relation to clinical trials. Target processing timeframes for clinical trials currently consist of approximately 30 business days for HREC review, followed by 5–7 days for TGA CTNs or 40 days for TGA CTAs. As the situation currently stands, the legislated timeframe for granting OGTR licenses is not reduced for clinical trials compared to TGA timeframes (it can still take up to 90 business days to obtain an OGTR license for a DNIR and 150 business days for an OGTR license for a DIR). The Third Review proposes improvements by further triaging regulatory processes, for example, by assigning applications to the new categories “full assessment,” “expedited assessment” or “permit approval” processes depending on risk (2). This is expected to improve timeframes, particularly for replication defective gene therapy viral vectors that have a long history of safe use with respect to the health and safety of people and the environment, such as AAV vectors. The TGA has also introduced reforms to improve timeframes and harmonization, for example, by introducing recognition of equivalent approvals by regulators in other jurisdictions and participating as a member of the Australia-Canada-Singapore-Switzerland-United Kingdom Access Consortium in promoting regulatory collaboration and alignment.

An outstanding issue affecting access is the lack of awareness among international sponsors of approval processes and timeframes in Australia. International sponsors often have authorization for a clinical trial under the FDA’s Investigational New Drug (IND) Scheme and assume that the local sponsor in Australia may only require HREC and TGA approvals. Surprises occur when it is belatedly discovered that an OGTR license is required and that the license holder needs to be an OGTR accredited organization. Although the accreditation and license applications can be submitted in parallel, it adds up to 90 business days to the approval processes. International sponsors should be encouraged to consider this early on in their regulatory strategy (e.g., before filing an IND) to ensure efficient access. The TGA, OGTR, and Australian biotechnology and trade organizations have a role in improving awareness of the Australian regulatory requirements for international sponsors.

Improvements to the patient journey will necessitate changes in how technology is used and how to translate between different regulatory systems. Agile responses to emerging health and market needs will facilitate access and manufacturing scaling up, out and back.

## Conclusion

Over the last 20 years, the Australian Gene Technology Regulatory Scheme has contributed to the advancement of gene-based therapeutics by providing a nationally consistent and transparent approach. The Scheme provides a clear set of classifications and one regulatory agency, the OGTR, which researchers, clinicians, and sponsors can turn to for expert advice. The Scheme is compatible with other applicable regulators such as the TGA. There are many regulatory challenges to address and further changes to the Scheme are planned to improve approval processes and make it more responsive to technological changes and harmonize processes between regulators. The OGTR licensing system has improved the governance of clinical studies, especially those conducted at multiple sites by placing responsibility for compliance on license holders and by ensuring appropriate risk management. The OGTR accreditation and facility certification systems have ensured clinical applications are sponsored by suitable organizations and conducted in appropriate containment facilities. Clinical trials are overseen by IBCs with appropriate expertise to assess safety of the gene modified products. Together these processes have ensured that organizations develop expertise and clinical and research capacity in the safe delivery of gene-based therapeutics. Implementation of recommendations from the Third Review of the Scheme is expected to further improve approval processes, timeframes, and access.

## Data

The information presented in this review is general in nature. There are exceptions to dealings and classifications, which may be assessed on a case-by-case basis by the OGTR.

Although some of the authors are OGTR staff members or members of the Gene Technology Technical Advisory Committee (GTTAC) and/or the Gene Technology Ethics and Community Consultative Committee (GTECCC), this paper is not intended as a source of advice (legal or otherwise) in relation to the Gene Technology Regulatory Scheme or risk management of GMO dealings. Instead, it provides an academic overview of the Scheme and its contribution to the safe advancement of research in Australia. Its content is not advice.

Although this paper mentions medications and brand names it is not promoting any medications or brands.

## Author Contributions

GO’S: acquisition and analysis of data, writing, review/editing, and coordination. JP, HM, and MD: acquisition and analysis of data and writing and review/editing. JR: conception, outline, and review/editing. All authors contributed to the article and approved the submitted version.

## Author Disclaimer

The views expressed in the article are those of the authors alone and do not represent an official view of an Australian Government, the Gene Technology Regulator, New South Wales Health, Sydney Local Health District or Royal Prince Alfred Hospital.

## Conflict of Interest

JR and GO’S are employed by the Royal Prince Alfred Hospital (RPAH) Sydney Local Health District (SLHD). MD, HM, and JP are employed by the Office of the Gene Technology Regulator. JR has served on the Gene Technology Technical Advisory Committee (GTTAC) since 2001, including as Chair from 2008 to the present. GO’S has served on GTTAC since 2011, including as cross-member of the Gene Technology Ethics and Community and Consultative Committee from 2015 to the present. JR has also served on the RPAH Institutional Biosafety Committee as Chair, on the TGA Advisory Committee on Biologicals and the NHMRC Mitochondrial Donation Expert Working Committee. He was President of the International Society for Cell and Gene Therapy (ISCT) 2018–20 and serves on the ISCT Board 2016–2022 which has links to many companies and affiliations with diverse organizations with interests in medical gene technologies. As Head of Department, Cell and Molecular Therapies, RPAH, and as Head of the Gene and Stem Cell Program at the Centenary Institute, he is responsible for undertaking investigator- and pharma-sponsored clinical trials of immunotherapies, cellular therapies, and gene therapies at RPAH and basic research at Centenary Institute often in collaboration with biotech and infrastructure companies; and has had Material Transfer Agreements or consultancy or honoraria with a number of companies, including Gilead, Novartis, Bluebird Bio, SPARK therapeutics, Cynata, Pfizer Inc., Atara, Bayer, and CRISPR Therapeutics. Any patents are held by SLHD, Centenary Institute or the University of Sydney as his primary affiliations. JR has research and clinical interests in gene technology, especially in hemophilia and thalassemia gene therapy, CAR T-cells and iPSCs. GO’S is also Executive Officer of the RPAH Institutional Biosafety Committee and Co-Chair of the Australia and New Zealand Legal and Regulatory Affairs Committee of ISCT.

## Publisher’s Note

All claims expressed in this article are solely those of the authors and do not necessarily represent those of their affiliated organizations, or those of the publisher, the editors and the reviewers. Any product that may be evaluated in this article, or claim that may be made by its manufacturer, is not guaranteed or endorsed by the publisher.
